# A systematic review of the cost and cost-effectiveness studies of immune checkpoint inhibitors

**DOI:** 10.1186/s40425-018-0442-7

**Published:** 2018-11-23

**Authors:** Vivek Verma, Tanja Sprave, Waqar Haque, Charles B. Simone, Joe Y. Chang, James W. Welsh, Charles R. Thomas

**Affiliations:** 1 Department of Radiation Oncology, Allegheny General Hospital, 320 East North Ave, Pittsburgh, PA 15212 USA; 2 Department of Radiation Oncology, University Hospital Heidelberg, Heidelberg, Germany; 3 Department of Radiation Oncology, Houston Methodist Hospital, Houston, TX USA; 4 Department of Radiation Oncology, University of Maryland Medical Center, Baltimore, MD USA; 5 Department of Radiation Oncology, University of Texas M.D. Anderson Cancer Center, Houston, TX USA; 6 Department of Radiation Oncology, Oregon Health & Science University, Portland, OR USA

**Keywords:** Cost-effectiveness, Value, Immunotherapy, Immune checkpoint inhibitor, Public policy, Public health, Health policy

## Abstract

**Background:**

Escalating healthcare costs are necessitating the practice of value-based oncology. It is crucial to critically evaluate the economic impact of influential but expensive therapies such as immune checkpoint inhibitors (ICIs). To date, no systematic assessment of the cost-effectiveness (CE) of ICIs has been performed.

**Methods:**

PRISMA-guided systematic searches of the PubMed database were conducted. Studies of head/neck (*n* = 3), lung (*n* = 5), genitourinary (*n* = 4), and melanoma (*n* = 8) malignancies treated with ICIs were evaluated. The reference willingness-to-pay (WTP) threshold was $100,000/QALY.

**Results:**

Nivolumab was not cost-effective over chemotherapy for recurrent/metastatic head/neck cancers (HNCs). For non-small cell lung cancer (NSCLC), nivolumab was not cost-effective for a general cohort, but increased PD-L1 cutoffs resulted in CE. Pembrolizumab was cost-effective for both previously treated and newly-diagnosed metastatic NSCLC. For genitourinary cancers (GUCs, renal cell and bladder cancers), nivolumab and pembrolizumab were not cost-effective options. Regarding metastatic/unresected melanoma, ipilimumab monotherapy is less cost-effective than nivolumab, nivolumab/ipilimumab, and pembrolizumab. The addition of ipilimumab to nivolumab monotherapy was not adequately cost-effective. Pembrolizumab or nivolumab monotherapy offered comparable CE profiles.

**Conclusions:**

With limited data and from the reference WTP, nivolumab was not cost-effective for HNCs. Pembrolizumab was cost-effective for NSCLC; although not the case for nivolumab, applying PD-L1 cutoffs resulted in adequate CE. Most data for nivolumab and pembrolizumab in GUCs did not point towards adequate CE. Contrary to ipilimumab, either nivolumab or pembrolizumab is cost-effective for melanoma. Despite these conclusions, it cannot be overstated that careful patient selection is critical for CE. Future publication of CE investigations and clinical trials (along with longer follow-up of existing data) could substantially alter conclusions from this analysis.

## Introduction

Immunotherapies have rapidly emerged as important tools in the oncologic arsenal. Although encompassing a diverse array of agents that act on the anti-tumoral immune system (e.g. monoclonal antibodies, small molecules, tumor vaccines, and viral or cellular therapies), the most frequently utilized immunotherapies are immune checkpoint inhibitors (ICIs). As of the writing of this article, there are six ICIs approved by the United States Food and Drug Administration (FDA) [1]: the programmed cell death-1 (PD-1) inhibitors nivolumab and pembrolizumab; the programmed cell death ligand-1 (PD-L1) inhibitors atezolizumab, durvalumab, and avelumab; and the cytotoxic T-lymphocyte-associated antigen-4 (CTLA-4) inhibitor ipilimumab. These agents have become the standard of care in appropriate clinical circumstances on the basis of numerous clinical trials demonstrating outcome improvements in recurrent and/or metastatic melanoma, non-small cell lung cancer (NSCLC), head and neck cancers (HNCs), and genitourinary cancers (GUCs) [2–17].

However, the primary drawback of ICIs are in the financial realm; this is especially important given that, for example, cancer care in the United States is expected to reach $173 billion by 2020 [18]. As a result, the shift to value-based oncology (VBO) is becoming increasingly apparent [19]. Moreover, because the current pace of oncologic expenditure may not be sustainable, the cost-effectiveness (CE) of these new and expensive therapies becomes essential to address.

Assessment of CE commonly occurs by means of modeling studies comparing two or more cohorts undergoing different interventions. After assembling the appropriate hypothetical population, numerous parameters are entered into the model, not limited to costs of primary and/or secondary therapy, probabilities of remaining disease/progression-free (usually based on established clinical data), probabilities of toxicities (with according management costs), and expected clinical outcomes until death (also based on clinical data and/or extrapolation thereof). Costs and outcomes are compiled for both groups for the desired modeling duration (referred to as the time horizon); arms are then compared for total costs, quality-adjusted life-years (QALYs), and the costs associated with each gained QALY (termed the incremental cost-effectiveness ratio (ICER)). The ICER denotes how much payment is required for one additional year of (quality-weighted) life and is compared with a pre-determined “willingness-to-pay” (WTP) threshold that differs by publication, society, economic system, time period, and other factors (most common thresholds range from $50,000–$150,000/QALY). Comparison of the ICER with the WTP threshold concludes whether or not the intervention is deemed “cost-effective”. Lastly, because input parameters are often estimated and/or extrapolated, extensive sensitivity analyses are performed to measure changes in costs and CE by varying any number of input parameters.

The two most common modeling methodologies are state-transition models (the most common subset of which is a Markov model) and partitioned survival (PS) models (a complete discussion regarding the nuances of each approach is beyond the scope of this systematic review and is provided elsewhere [20]). Markov modeling is more flexible and can include an infinite number of health states but can involve greater uncertainty from more modeling assumptions (e.g. probabilities in transitioning from one health state to the next). PS models, although less flexible in terms of allowable health states, involve fewer assumptions and rely on inputted Kaplan-Meier/extrapolated survival figures rather than the aforementioned transition probabilities; however, that increased reliance may result in distortion if “actual” survival deviates from the extrapolation. Nevertheless, it has been postulated that for many oncologic studies with three basic health states (progression-free/stable disease, progression, and death) and longer-term survival data requiring comparatively less extrapolation, results from both models can be quite comparable [20].

The current global healthcare climate is rapidly evolving. As a result, it is crucial to critically evaluate the economic impact of influential but expensive therapies, especially given the mounting costs of oncologic care and the escalating necessity to practice VBO. This work, the first known comprehensive review of CE analyses pertaining to ICI therapy, has major implications on health policy, public policy, VBO, and ongoing ICI clinical trials.

## Materials and methods

This systematic review was conducted using the Preferred Reporting Items for Systematic Reviews and Meta-Analyses (PRISMA) guidelines [21] and paralleled the methodology of existing CE-related systematic reviews [22]. Eligibility criteria were published studies in the English language evaluating CE of any of the six currently FDA-approved ICIs. The PubMed database was the primary data source, as well as publications found from references of selected articles and studies known to the authors. Unpublished abstracts were not included owing to the inability to completely assess validity and methodologies. Searches were intended to identify all articles addressing this subject with any of the following search terms: cost, cost-effectiveness, value, economics, policy, monetary, reimbursement, insurance; immunotherapy, immune checkpoint inhibitor, nivolumab, pembrolizumab, atezolizumab, durvalumab, avelumab, and ipilimumab. Care was taken to ensure that the inclusion criteria were sufficiently broad, so that possibly pertinent publications could be better appraised by individual screening rather than being excluded by the initial search. Systematic searches did not utilize date restrictions and included articles published through April 1, 2018. It was not possible to perform a meta-analysis on the available literature owing to the inherent heterogeneity in scope, study comparisons, and study designs.

**Fig. 1:**
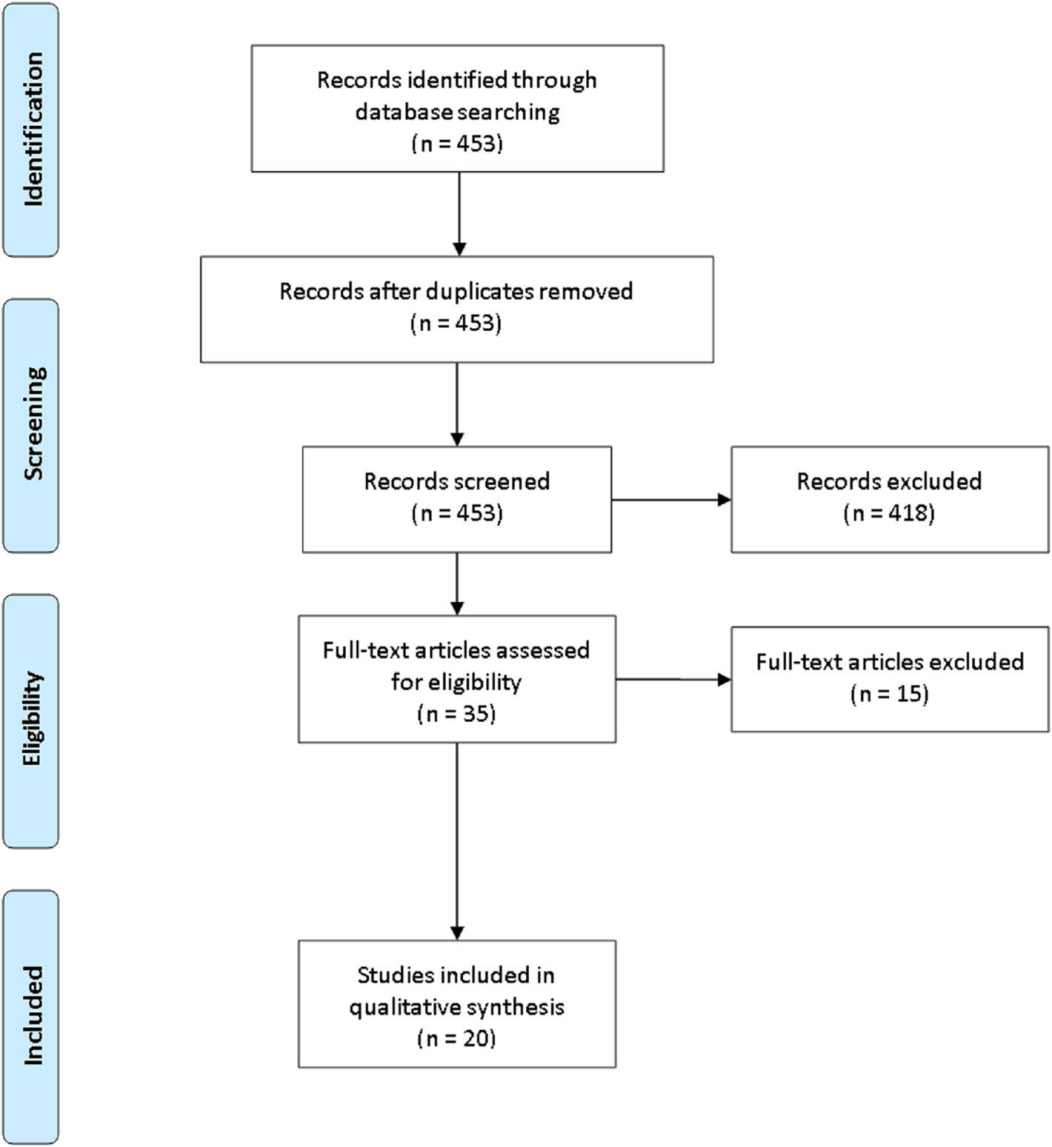
PRISMA diagram illustrating systematic searches of this review

Based on the initial searches, a total of 453 articles were identified, which were independently screened for the inclusion criteria (Fig. 1). Of these, 418 were determined to be ineligible, largely owing to transient references to CE without specific analyses, along with non-original research (e.g., letters to the editor or commentaries). Of the 35 publications remaining, 15 were further eliminated; eight were unpublished abstracts and five were review articles. Two publications were ultimately removed owing to non-modeling and/or non-comparative designs that evaluated cost but not specifically CE [23, 24]. Thus, 20 original investigations were found to have sufficient focus and relevance to be incorporated into the systematic review.

The reference WTP threshold chosen was $100,000/QALY because this was the most common value among the 20 publications, although evaluation with other thresholds is further elaborated upon in the Discussion.

## Results

### Head and neck cancers

Table 1 displays a summary of three Markov modeling studies evaluating the CE of nivolumab for recurrent or metastatic HNCs. A report from investigators at Cleveland Clinic compared nivolumab with “standard” therapy, defined as clinician’s choice of cetuximab, docetaxel, or methotrexate (per the CheckMate 141 trial) [25]. Using the latter group as a reference, nivolumab cost $140,672/QALY; when applying PD-L1 testing with a cutoff of ≥1%, the ICER was minimally changed ($131,066/QALY). When evaluating the CE of nivolumab over individual agents, the ICER relative to cetuximab was $89,786/QALY; however, the ICERs relative to methotrexate and docetaxel were considerably higher at $154,411/QALY and $154,191/QALY, respectively. Nivolumab was thus not concluded to be a cost-effective option.

**Table 1:** Details regarding cost-effectiveness studies of head and neck cancers

Reference, Country, Year	Comparison	Methodology^a^	Costs	QALYs	ICER	WTP	Conclusions	Criticisms
Ward et al., USA, 2017 [25]	Nivo vs. standard (choice of cetux, doc, or MTX) for recurrent/metastatic HNC	Markov; PD-L1 cutoff ≥1%; accounted for toxicity, administration, end-of-life costs	$73,463 nivo, $26,133 standard	0.626 nivo, 0.289 standard	Relative to standard, nivo $140,672/QALY; nivo with PD-L1 testing $131,066/QALY; relative to cetux, nivo $89,786/QALY; relative to MTX, nivo $154,411/QALY; relative to doc, nivo $154,191/QALY	$100,000/QALY	Nivo not CE in this setting; PD-L1 testing minimally influences results	- Although per prospective data, heterogeneity of “standard” cohort using several different agents difficult to interpret, and not necessarily representative of practice patterns - Disutility scores based on weaker, non-prospective data - Lack of accountability for grade < 3 toxicities or p16 disease
Zargar et al., Canada, 2018 [26]	Nivo vs. doc for recurrent/metastatic HNC	Markov; accounted for PD-L1, toxicity, and end-of-life costs	CAD 60,035 ($46,563) nivo, CAD 41,212 ($31,964) doc	0.248 nivo, 0.130 doc	Relative to doc, nivo CAD 144,744 ($112,263)/QALY	CAD 100,000 ($78,385)/QALY	Although numerically more favorable in younger, p16+, PD-L1 > 5% patients, nivo not CE	- Although prospective trial data used, that trial did not utilize single-agent docetaxel as in this study, but rather a combination of three agents - Did not account for administration costs - Only grade ≥ 3 toxicities accounted for, as a one-time cost
Tringale et al., USA, 2018 [27]	Nivo vs. standard (choice of cetux, doc, or MTX) for recurrent/metastatic HNC	Markov; accounted for toxicity, administration, societal, and end-of-life costs	$174,800 nivo, $57,000 standard	0.796 nivo, 0.396 standard	Relative to standard, nivo $294,400/QALY; relative to cetux, nivo $182,200/QALY	$100,000/QALY	Nivo not CE in this setting	- Overall modeling horizon of 30 years, when exceedingly low numbers of patients still alive, causing errors in survival extrapolation and thus costs - Only grade ≥ 3 toxicities accounted for, as a one-time cost - Did not consider PD-L1 status or p16 disease

*QALY* quality-adjusted life year, *ICER* incremental cost-effectiveness ratio, *WTP* willingness to pay (threshold); nivo, nivolumab; cetux, cetuximab; doc, docetaxel, *MTX* methotrexate, *HNC* head and neck cancers, *CE* cost-effective, *PD-L1* programmed cell death ligand-1, *CAD* Canadian dollar

^a^All studies consisted of three basic health states (progression-free (stable), progressive disease, and death); all studies performed sensitivity analyses in addition to the base case

Zargar and colleagues performed a study evaluating nivolumab versus docetaxel, the only such study to evaluate PD-L1 cutoff values and p16 status on subgroup analysis [26]. The authors measured an ICER of CAD (Canadian dollars) 144,744 ($112,263)/QALY for nivolumab as compared to docetaxel. Importantly, the ICER numerically decreased as the PD-L1 cutoff increased and the proportion of younger and/or p16+ patients were evaluated; however, nivolumab was still not deemed cost-effective at a WTP threshold of $100,000/QALY.

A final study assessed nivolumab against “standard” therapy as above [27]. Although not accounting for PD-L1 or p16 status, total costs were $174,800 and $57,000, respectively; corresponding QALYs were 0.796 and 0.396. This amounted to an ICER of $294,300/QALY, which was not cost-effective.

### Non-small cell lung cancer

Details of the pertinent studies are presented in Table 2. A study from Canadian investigators notably utilized both PS and Markov modeling to examine nivolumab versus docetaxel versus erlotinib for recurrent disease [28]. Of note, the authors observed negligible cost and CE estimates between both modeling approaches. The ICER for nivolumab relative to docetaxel was CAD 152,229 ($117,857)/QALY, and relative to erlotinib was CAD 141,838 ($109,811)/QALY. Although a particular WTP value was not mentioned, these values indicated that nivolumab could be considered cost-effective at higher WTP thresholds (e.g. $150,000/QALY), but it was not so at a $100,000/QALY threshold.

**Table 2:** Details regarding cost-effectiveness studies of non-small cell lung cancer

Reference, Country, Year	Comparison	Methodology^b^	Costs	QALYs	ICER	WTP	Conclusions	Criticisms
Goeree et al., Canada, 2016 [28]	Nivo vs. doc vs. erl for recurrent stage IIIB/IV SCC	PS^a^ + Markov; accounted for toxicity, administration, and end-of-life costs	CAD 139,016 ($107,631) nivo, CAD 38,812 ($30,049) doc, CAD 39,920 ($30,906) erl	1.23 nivo, 0.58 doc, 0.54 erl	Relative to doc, nivo CAD 152,229 ($117,857)/QALY and relative to erl, 141,838 ($109,811)/QALY	No specific amount in Canada	Compared with doc or erl, nivo may or may not be CE depending on WTP threshold	- Overall modeling horizon of 10 years, when low numbers of patients still alive, causing errors in survival extrapolation and thus costs - Difficulty in interpreting next-line therapies - Only grade ≥ 3 toxicities accounted for, as a one-time cost
Matter-Walstra et al., Switzerland, 2016 [29]	PD-L1 testing + subsequent decision vs. nivo vs. doc for recurrent non-SCC	Markov; PD-L1 testing cutoffs ≥1% and ≥ 10%; accounted for end-of-life costs and reducing nivo dose and duration	CHF 37,618 ($39,378) doc, CHF 66,208 ($69,306) nivo, CHF 47,410 ($49,628) nivo with dose reduction, CHF 55,394 ($57,992) with duration reduction	0.53 doc, 0.69 nivo	Relative to doc, nivo CHF 177,478 ($185,802)/QALY, reduced dose CHF 60,787 ($63,638)/QALY, reduced duration CHF 110,349 ($115,524)/QALY	CHF 100,000 ($104,690) /QALY	Although not at baseline, nivo is CE by dose reduction and increased PD-L1 threshold	- Overall modeling horizon of complete lifetime, causing errors in survival extrapolation and thus costs - In addition to lack of a discount rate, did not account for most toxicities, administration, or death costs - Median PFS favored doc, but 1-year PFS favored nivo; difficult to account for in model
Aguiar et al., USA, 2017 [30]	Nivo, pembro, or atezo ± prior PD-L1 testing vs. doc for recurrent SCC/non-SCC	Decision-analytic model; PD-L1 testing cutoffs ≥1%/≥5%/≥10% for nivo and ≥ 1%/50% for pembro; accounted for administration, monitoring, end-of-life costs	$140,453 nivo for SCC and $100,791 nivo for non-SCC (PD-L1 untested), $82,201 pembro (PD-L1 ≥ 1%), $122,155 atezo (PD-L1 untested); doc $39,516–$48,182	0.82 nivo (SCC), 0.87 nivo (non-SCC), 0.92 pembro, 0.90 atezo, 0.54–0.59 doc	Relative to doc, nivo $155,605/QALY (SCC) and nivo $187,685/QALY (non-SCC), pembro $98,421/QALY, atezo $215,802/QALY	$100,000/QALY	Atezo not CE; pembro is CE; although not at baseline, nivo is CE by increased PD-L1 threshold	- Toxicity types and one-time costs thereof not explained - Unclear whether patients treated until PD, along with details of next-line therapy (if present) - Analysis and reflections based on USA Medicare system, limiting broader applicability
Huang et al., USA, 2017 [31]	Pembro vs. doc for recurrent non-SCC	PS; PD-L1 testing cutoff ≥50%; accounted for toxicity, administration, end-of-life costs	$136,921 doc, $297,443 pembro	0.76 doc, 1.71 pembro	Relative to doc, pembro $168,619/QALY	Per capita GDP × 3	Pembro CE at the particular WTP threshold, which is nonstandard and thus questionable	- Overall modeling horizon of 20 years, when exceedingly low numbers of patients still alive, causing errors in survival extrapolation, which was done through retrospective population datasets - Survival found to most influence costs; thus, extrapolation may markedly influence overall costs - Only grade ≥ 3 toxicities accounted for, as a one-time cost
Huang et al., USA, 2017 [31]	Pembro vs. several types of chemo for first-line SCC/non-SCC	PS; PD-L1 testing cutoff ≥50%; accounted for toxicity, administration, end-of-life costs	$260,223 chemo, $362,662 pembro	1.55 chemo, 2.60 pembro	Relative to chemo, pembro $97,621/QALY	No specific amount used	Pembro is CE in this setting	- Overall modeling horizon of 20 years, when exceedingly low numbers of patients still alive, causing errors in survival extrapolation (done through retrospective population datasets), which may impact costs secondarily - Heterogeneity in treating with variety of chemo - Only grade ≥ 3 toxicities accounted for, as a one-time cost

*QALY* quality-adjusted life year, *ICER* incremental cost-effectiveness ratio, *WTP* willingness to pay (threshold); nivo, nivolumab; doc, docetaxel; erl, erlotinib; *SCC* squamous cell carcinoma, *PS* partitioned survival, *CAD* Canadian dollar, *CE* cost-effective, *PFS* progression-free survival, *OS* overall survival, *PD-L1* programmed cell death ligand-1, *CHF* Swiss francs

^a^Data for partitioned survival model not shown owing to virtual similarity as Markov data

^b^All studies consisted of three basic health states (progression-free (stable), progressive disease, and death); all studies performed sensitivity analyses in addition to the base case

Unlike the previous study, a Swiss Markov analysis of recurrent NSCLC incorporated PD-L1 testing [29]. There were three cohorts: nivolumab (without PD-L1 testing) versus docetaxel versus up-front PD-L1 testing. The latter arm encompassed a decision-based step dependent on the PD-L1 result; if the PD-L1 value was above the given cutoff (both ≥1% and ≥ 10% were utilized), nivolumab was administered (all others received docetaxel). Under this model, up-front nivolumab was not cost-effective (CHF (Swiss francs) 177,478 ($185,802)/QALY) over docetaxel. However, the authors importantly elucidated that among patients with PD-L1 ≥ 1%, not only was nivolumab associated with a strikingly lower ICER (CHF 65,774 ($68,443)/QALY), administration of docetaxel in that setting was not cost-effective (CHF 133,267 ($138,675)/QALY). Increasing the cutoff to ≥10% resulted in the nivolumab ICER to further decrease (CHF 37,860 ($39,396)/QALY). Lastly, this is one of the few CE studies to evaluate the impact of ICI dose and/or duration reductions. Although a major assumption was made that reducing the dose (from 3 mg/kg to 1 mg/kg) and duration (until progression to 3 months maximum) would yield similar efficacy, doing so improved the CE profile of nivolumab. Although the ICER for reduced duration (CHF 110,349 ($115,524)/QALY) was above the CHF 100,000 ($104,690)/QALY WTP cutoff, dose reduction would result in a cost-effective ICER (CHF 60,787 ($63,638)/QALY). Despite these substantial findings, there were notable limitations such as limited consideration of most toxicities and drug administration costs; this was the only study that also did not apply an annual discount rate to account for changes in the value of money over time.

Similar conclusions regarding PD-L1 testing were conveyed by Aguiar et al., who evaluated nivolumab, pembrolizumab, and atezolizumab against docetaxel for recurrent NSCLC [30]. Pembrolizumab was cost-effective ($98,421/QALY), whereas atezolizumab was not ($215,802/QALY). Although nivolumab was not cost-effective at baseline, increasing the PD-L1 threshold improved its CE profile. Although performing this measure for squamous disease did not result in superior CE using a WTP of $100,000/QALY ($201,461/QALY for ≥1%, $135,080/QALY for ≥5%, $131,159/QALY for ≥10%), this was observed for non-squamous cases ($112,311/QALY for ≥1%, $72,897/QALY for ≥5%, $78,921/QALY for ≥10%).

Huang and coworkers performed PS modeling to evaluate the CE of pembrolizumab (PD-L1 cutoff ≥50%) versus docetaxel for recurrent disease [31]. The ICER for the former as compared to the latter was $168,619/QALY; although the authors deemed pembrolizumab cost-effective, the WTP value utilized was questionable (three times the per capita gross domestic product). Notable, the high ICER was likely related to the high overall cost of pembrolizumab ($297,443), which is potentially related to a proportion of patients allowed to continue therapy even after disease progression.

The same group utilized similar methodologies in the setting of newly-diagnosed metastatic disease [32]. In this analysis, pembrolizumab (PD-L1 cutoff ≥50%) was cost-effective with an ICER of $97,621/QALY. However, the reference group was a heterogeneous collection of chemotherapy agents (not docetaxel alone), which may limit interpretation.

### Genitourinary cancers

Table 3 illustrates the four studies evaluating ICIs for GUCs. Wan and coworkers conducted the first of three studies to assess nivolumab versus everolimus for recurrent renal cell carcinoma (RCC) [33]. Using a PS model, they determined that nivolumab cost $151,676/QALY relative to everolimus, which was not cost-effective. It is possible, however, that the results could have been somewhat different had end-of-life costs been incorporated.

**Table 3:** Details regarding cost-effectiveness studies of genitourinary cancers

Reference, Country, Year	Comparison	Methodology^a^	Costs	QALYs	ICER	WTP	Conclusions	Criticisms
Wan et al., USA, 2017 [33]	Nivo vs evero for recurrent RCC	PS; accounted for toxicity and administration costs	$211,407 nivo, $167,405 evero	1.79 nivo, 1.5 evero	Relative to evero, nivo $151,676/QALY	$100,000/QALY	Nivo not CE in this setting	- Overall modeling horizon of 20 years, when low numbers of patients still alive, causing errors in survival extrapolation and thus costs - Only grade ≥ 3 toxicities (*n* = 3) accounted for, as a one-time cost - Lack of accountability for hospice/palliative care and death costs
McCrea et al., USA, 2018 [34]	Nivo vs evero for recurrent RCC	PS; accounted for toxicity, administration, follow-up, and end-of-life costs	$197,089 nivo, $163,902 evero	2.79 nivo, 2.15 evero	Relative to evero, nivo $51,714/QALY	$150,000/QALY	Nivo is CE in this setting	- Overall modeling horizon of 25 years, when exceedingly low numbers of patients still alive, causing errors in survival extrapolation and thus costs - Uncertainty of whether proportion of patients treated past time of progression - Assumed non-progressors at 22 months had same utility as responders
Sarfaty et al., USA, 2018 [35]	Nivo vs evero vs placebo for recurrent RCC	Markov; accounted for toxicity and administration costs	$101,070 nivo, $50,935 evero	Nivo 0.34 QALYs higher than evero/placebo	Relative to evero, nivo $146,532/QALY; relative to placebo, nivo $226,197/QALY	$100–150,000/QALY	Nivo not CE over placebo, but cost-effectiveness vs. evero depends on particular WTP	- Overall modeling horizon of 10 years, when low numbers of patients still alive, causing errors in survival extrapolation and thus costs - Only grade ≥ 3 toxicities accounted for, as a one-time cost - Lack of accountability for hospice/palliative care and death costs
Sarfaty et al., USA, 2018 [35]	Pembro vs taxanes for recurrent bladder cancer	Markov; accounted for toxicity and administration costs	Cost of pembro $44,325 higher than taxanes	Pembro 0.36 QALYs higher than taxanes	Relative to taxanes, pembro $122,557/QALY	$100–150,000/QALY	Cost-effectiveness of pembro depends on particular WTP	- Lack of accountability for patients receiving taxanes that subsequently undergo pembro - Only grade ≥ 3 toxicities accounted for, as a one-time cost - Lack of accountability for hospice/palliative care and death costs

*QALY* quality-adjusted life year, *ICER* incremental cost-effectiveness ratio, *WTP* willingness to pay (threshold); nivo, nivolumab; evero, everolimus, *RCC* renal cell carcinoma; PS, partitioned survival, *CE* cost-effective

^a^All studies consisted of three basic health states (progression-free (stable), progressive disease, and death); all studies performed sensitivity analyses in addition to the base case. No study evaluated programmed cell death ligand-1 status

The results of the aforementioned study are contrary to those of McCrea et al., who also utilized PS methodology [34]. However, the ICER therein was just $51,714/QALY; given that overall costs were similar between studies, a likely factor relates to different survival extrapolation methods in each study, leading to dissimilar QALYs (both studies extrapolated survival for 20–25 years, which may amplify extrapolation errors between studies).

A third study performed the same comparison (with a Markov approach) but also adding a placebo group [35]. The QALY differential between groups was similar to Wan et al., and the ICER of nivolumab was accordingly similar at $146,532/QALY. Although the authors acknowledged that this figure may be considered cost-effective at a WTP value of $150,000/QALY (but not $100,000/QALY), a notable observation was that nivolumab was not more cost-effective than placebo (ICER $226,197/QALY).

The same group performed the first known CE (Markov) analysis for recurrent bladder cancer and compared pembrolizumab with taxanes [36]. Although PD-L1 levels were not accounted for, the ICER for pembrolizumab was $122,557/QALY, concluding that the compound would be deemed cost-effective at higher WTP thresholds, but not at lower values. Of note, the ICER was compared to WTP thresholds in Australia, the United Kingdom, and Canada, none of which would have resulted in adequate CE.

### Melanoma

A summary of the relevant publications for metastatic/unresected melanoma can be found in Table 4. The first study utilized a Markov approach to compare ipilimumab and best supportive care [37]. Because the latter term did not include chemotherapy, its applicability is limited as a reference group; nevertheless, ipilimumab was associated with an ICER of $128,656/QALY. Although not cost-effective at a WTP definition of $100,000/QALY, the authors utilized a $146,000/QALY value and thus labeled ipilimumab as economically appropriate.

**Table 4:** Details regarding cost-effectiveness studies of melanoma

Reference, Country, Year	Comparison	Methodology^a^	Costs	QALYs	ICER	WTP	Conclusions	Criticisms
Barzey et al., USA, 2013 [37]	Ipi vs. BSC for recurrent/metastatic disease	Markov; accounted for toxicity and administration costs	$168,602 ipi, $21,886 BSC	1.76 ipi, 0.62 BSC	Relative to BSC, ipi $128,656/QALY	$146,000/QALY	Ipi CE using given WTP threshold, not so at more accepted cutoffs	- Overall modeling horizon of complete lifetime, causing errors in survival extrapolation and thus costs - Lack of accountability for hospice/palliative care and death costs - BSC arm (without chemotherapy) provides little meaningful clinical comparison
Curl et al., USA, 2014 [38]	Dac vs. vem vs. vem + ipi for unresected/metastatic BRAF mutant disease	Deterministic expected-value model; accounted for toxicity, administration, and follow-up costs	$8391 dac, $156,831 vem, $254,695 vem + ipi	0.30 dac, 0.72 vem, 1.34 vem + ipi	Relative to dac, vem $353,993/QALY, vem + ipi $158,139/QALY	No specific amount used	Vem or vem + ipi not CE in this setting	- Overall modeling horizon of complete lifetime, causing errors in survival extrapolation and thus costs - Assumed effect of vem + ipi is seamlessly additive - Lack of accountability for hospice/palliative care and death costs; unclear methodology for toxicity costs
Bohensky et al., Australia, 2016 [39]	Nivo vs. ipi for unresected/metastatic BRAF WT disease	Markov; accounted for toxicity, administration, and end-of-life costs	$178,612 nivo, $138,987 ipi	2.5 nivo, 1.2 ipi	Relative to ipi, nivo $30,475/QALY	$35,000/QALY	Nivo is more CE than ipi in this setting	- Overall modeling horizon of 10 years, causing errors in survival extrapolation and thus costs - Used data from second-line ipi and extrapolated to first-line ipi - Assumed patients weigh same as mean Australian body weight in trial (dosed accordingly); duration of therapy assumed to be same as the mean amount on trial
Oh et al., USA, 2017 [40]	Nivo vs. ipi vs. nivo+ipi for unresected/metastatic disease	Markov; accounted for toxicity, administration, follow-up, and end-of-life costs	$169,320 nivo, $213,763 ipi, $228,352 both	4.24 nivo, 3.68 ipi, 4.37 both	Relative to nivo, ipi was dominated; relative to ipi, both $21,143/QALY; relative to nivo, both $454,092/QALY	$100,000/QALY	Nivo (single-agent) is most CE in this setting; PD-L1 status changes cost-effectiveness negligibly	- Overall modeling horizon of 14.5 years, causing errors in survival extrapolation and thus costs - Owing to no overall survival data, survival figures were dependent on progression-free survival values only - Did not account for 2nd or 3rd line therapies
Wang et al., USA, 2017 [41]	Pembro vs. ipi for unresected/metastatic disease	PS; accounted for toxicity, administration, follow-up, and end-of-life costs	$303,505 pembro, $239,826 ipi	3.45 pembro, 2.67 ipi	Relative to ipi, pembro $81,091/QALY	$100,000/QALY	Pembro is more CE than ipi in this setting	- Overall modeling horizon of 20 years, causing errors in survival extrapolation and thus costs - Assumed no systemic therapy of any kind following progression - Pembro planned for maximum of 24 months in model (instead of until progression)
Miguel et al., Portugal, 2017 [42]	Pembro vs. ipi for unresected/metastatic disease	PS; accounted for toxicity, administration, and end-of-life costs	€156,268 ($191,924) pembro, €110,034 ($135,140) ipi	3.31 pembro, 2.33 ipi	Relative to ipi, pembro €47,221 ($57,988)/QALY	€50,000 ($61,407)/QALY	Pembro is more CE than ipi in this setting	- Overall modeling horizon of 40 years, when exceedingly low numbers of patients still alive, causing errors in survival extrapolation and thus costs - Only grade ≥ 3 toxicities accounted for, as a one-time cost - Pembro planned for maximum of 24 months in model (instead of until progression)
Kohn et al., USA, 2017 [43]	Dac vs. nivo vs. ipi vs. nivo+ipi vs. pembro (q2w) vs. pembro (q3w) for unresected/metastatic disease	Markov with built-in transition to 2nd and 3rd line therapies; accounted for toxicity, administration, and end-of-life costs	$146,775 dac, $172,219 nivo, $152,403 ipi, $206,435 nivo+ipi, $164,871 q2w pembro, $127,626 q3w pembro	0.26 dac, 0.54 nivo, 0.34 ipi, 0.56 nivo+ipi, 0.43 q2w pembro, 0.38 q3w pembro	Relative to q3w pembro, dac, ipi, and q2w pembro were dominated; nivo $66,800/QALY; nivo+ipi $319,723/QALY	$100,000/QALY	Nivo or q3w pembro (followed by 2nd line ipi) is most CE in this setting	- Overall modeling horizon of complete lifetime, causing errors in survival extrapolation and thus costs - No prospective data for several arms (e.g. pembro followed by 2nd line ipi) - Did not use immunotherapy dosing by body weight; although ongoing trials may not utilize weight-based dosing, previous trials (i.e., major sources of extracted data) have done so
Meng et al., England, 2018 [44]	Dac vs. ipi vs. nivo for unresected/metastatic BRAF WT; ipi vs. dab vs. vem vs. nivo for BRAF mutant disease	Markov; accounted for toxicity, administration, and end-of-life costs	BRAF WT: dac £25,228 ($35,542), ipi £57,158 ($80,532), nivo £97,898 ($137,931); BRAF mutant: ipi £56,621 ($79,775), dab £71,511 ($100,754), vem £74,001 ($104,262), nivo £88,228 ($124,307)	1.23 dac, 2.54 (avg) ipi, 1.69 dab, 1.70 vem, 4.29 (avg) nivo	BRAF WT: relative to dac, ipi £22,589 ($31,825)/QALY, nivo £24,483 ($34,493)/QALY; BRAF mutant: relative to ipi, dab and vem dominated; nivo £17,362 ($24,460)/QALY	£50,000 ($70,462)/QALY	Nivo is most CE in these settings	- Overall modeling horizon of complete lifetime, causing errors in survival extrapolation and thus costs - Model sensitive to treatment duration, but nonuniform comparison of continuing nivo for 2 years versus dac and vem until progression - Unclear description of cost summary with 2nd line of therapy

*QALY* quality-adjusted life year, *ICER* incremental cost-effectiveness ratio, *WTP* willingness to pay (threshold); ipi, ipilimumab, *BSC* best supportive care, *CE* cost-effective; dac, dacarbazine; vem, vemurafenib; nivo, nivolumab, *WT* wild-type; pembro, pembrolizumab, *PS* partitioned survival, *dab* dabrafenib; avg., average

^a^All studies but one (Kohn et al) consisted of three basic health states (progression-free (stable), progressive disease, and death); all studies performed sensitivity analyses in addition to the base case

A publication from the University of California San Francisco compared dacarbazine, vemurafenib, or vemurafenib/ipilimumab for BRAF-mutant disease [38]. Whereas overall prices for dacarbazine were low ($8391), costs were substantially higher for vemurafenib without ($156,831) or with ($254,695) ipilimumab. Accordingly, as compared to dacarbazine, neither group was cost-effective (vemurafenib $353,993/QALY, combined $158,139/QALY).

The initial report evaluating nivolumab (versus ipilimumab) was performed by Bohensky and colleagues [39]. This study of BRAF wild-type patients utilized Markov methodology; a large difference in QALYs (2.5 versus 1.2, respectively) likely led to the clearly superior CE of nivolumab ($30,475/QALY).

Another Markov analysis provided supportive data for the prior study [40]. By means of a Markov approach, this investigation compared nivolumab, ipilimumab, or both. Although combined therapy was evidently only slightly more expensive than ipilimumab alone ($228,352 versus $213,763), either nivolumab alone (no ICER because of dominance) or nivolumab/ipilimumab ($21,143/QALY) were superior to ipilimumab alone. However, combined therapy was not superior to nivolumab alone ($454,092/QALY). Of note, that study was the only melanoma investigation to evaluate PD-L1 levels, but did not find an appreciable CE changes based on this variable.

Two investigations evaluated pembrolizumab versus ipilimumab using PS-based approaches [41, 42]. Despite the caveats of being from two different economic systems (United States and Europe), both found similar results in that pembrolizumab was more cost-effective than ipilimumab. The United States publication demonstrated an ICER of $81,091/QALY, as compared to €47,221 ($57,988)/QALY in the European study.

Kohn and colleagues performed a highly analytic Markov analysis of six arms, with a unique feature of analyzing a built-in transition to second and third-line therapies [43]. The six cohorts were as follows: dacarbazine, nivolumab, ipilimumab, nivolumab/ipilimumab, every two weeks (q2w) pembrolizumab, and q3w pembrolizumab. On base case analysis, dacarbazine, ipilimumab, and q2w pembrolizumab (accounting for receipt of second-line ICIs for each) were dominated. When comparing the remainder, with reference to q3w pembrolizumab, nivolumab was comparably cost-effective (ICER $66,800/QALY), but nivolumab/ipilimumab was unacceptably expensive (ICER $319,723/QALY).

The final investigation, performed in the United Kingdom, created two parallel Markov simulations of BRAF mutant and wild-type disease [44]. For the latter, dacarbazine was compared with ipilimumab as well as nivolumab; for the former, ipilimumab was compared with dabrafenib, vemurafenib, and nivolumab. In the BRAF wild-type comparison, both ipilimumab (£22,589 ($31,825)/QALY) and nivolumab (£24,483 ($34,493)/QALY) were cost-effective compared to dacarbazine. The BRAF mutant analysis (which utilized ipilimumab as a reference) revealed that although both BRAF inhibitors were dominated, nivolumab demonstrated appropriate CE (£17,362 ($24,460)/QALY).

## Discussion

Because costs of cancer care are rising at an unprecedented rate, it is essential to provide evidence-based justification to implement promising but expensive therapeutic approaches such as ICIs. This systematic assessment of CE publications demonstrates several important findings. First, nivolumab is not cost-effective for recurrent/metastatic HNCs. Next, pembrolizumab is cost-effective for recurrent/metastatic NSCLC; although not the case for nivolumab, applying PD-L1 cutoffs may result in adequate CE. Regarding GUCs, most data for nivolumab or pembrolizumab would not result in CE using a WTP threshold of $100,000/QALY. Contrary to ipilimumab, either nivolumab or pembrolizumab are cost-effective for melanoma.

Given that CE analyses are largely dependent on available randomized data, the overall conclusions of this study are logical. For instance, nivolumab demonstrated a statistically significant overall survival (OS) improvement in HNCs in the CheckMate 141 trial, but the absolute OS difference between groups was just 2.4 months; moreover, the progression-free survival (PFS) was actually numerically lower in the nivolumab arm [13]. This mirrors results from CheckMate 025 for metastatic RCC insofar as insignificant PFS differences, despite the more notable OS spread [9]. Although there are no randomized data comparing nivolumab versus pembrolizumab for melanoma, meta-analyses suggest comparable outcomes [45, 46]; furthermore, there are randomized data supporting the superiority of either of these agents over ipilimumab alone [4, 15].

The conclusions of this review as pertaining to NSCLC are also important to discuss, especially in context of PD-L1 testing. In the KEYNOTE-024 study of previously untreated NSCLC, pembrolizumab resulted in a statistically higher PFS and OS over platinum-based chemotherapy [12]; however, the analogous CheckMate 026 investigation with nivolumab showed no differences [47]. Although this may adequately explain the CE findings above, they must be contextualized by the fact that KEYNOTE-024 required a PD-L1 threshold of ≥50%, in contrast to the ≥1% utilized in CheckMate 026. Multiple CE studies corroborated that increasing the PD-L1 cutoff could make nivolumab cost-effective [29, 30]; in this sense, they imply that the enhanced CE of pembrolizumab is not from superiority of the drug itself, but rather the inherently sharpened patient selection by requiring a PD-L1 ≥ 50%. Because trials using nivolumab did not require such standards, it is not cost-effective for all patients; however, careful PD-L1-related patient selection could achieve adequate CE. Multiple meta-analyses have added credence to this notion, demonstrating similar outcomes with either agent when stratifying for PD-L1 values, collectively concluding that the PD-L1 value may be clinically more important than the particular agent [48, 49]. However, this notion remains to be collaborated in full publications of large randomized trials; to this extent, the interaction between PD-L1 levels and other candidate prognostic factors of the particular tumor type would be an area of further exploration.

Hence, there is an important lesson when measuring and interpreting CE: patient selection is arguably the single most important factor influencing the CE of a particular intervention. For instance, one may not expect many agents to be cost-effective for very elderly patients with poor performance status, because they could be less likely to experience outcome improvements and/or toxicity reductions from a particular intervention. Although PD-L1 is a clear example of the necessity to refine patient selection for expensive therapeutics, no analyzed study in GUCs and just one [40] in melanoma accounted for this parameter. However, the analysis by Zargar and colleagues on HNCs suggested (in addition to PD-L1) improved CE in younger patients with p16 disease [26]. It is hence highly encouraged that future CE analyses better evaluate subgroups for more optimal economic favorability.

Another factor that can substantially affect the perceived CE of an ICI compound is the particular WTP threshold utilized. There is no single value that has been adopted because WTP inherently depends on a multitude of factors. These include the particular society and economic system, as it is well-known that the United States has higher WTP cutoffs as compared to other countries [36]. Factors at the individual level may also be considered, because a particular individual could be willing to pay a different amount for one additional QALY than would his physician or insurance plan. Based on these notions, the debate of the “optimal” WTP threshold inherently involves an ethical component from balancing “the price of life” with economic sustainability; this ethical debate potentiates the ambiguity of “the ideal WTP value”. A review of the Tufts CE registry demonstrates that most studies use the historical $50,000/QALY threshold [50]. However, this value has been criticized for being antiquated and unadjusted for modern economic times [51]. For example, if the historical $50,000/QALY value was used herein, only three of the twenty ICI investigations analyzed would be deemed cost-effective (nivolumab for melanoma) [39, 40, 44], concluding that ICIs are not cost-effective for NSCLC, HNCs, or GUCs. The particular reference WTP threshold we chose to utilize was $100,000/QALY, simply because this was the most common value in the 20 studied publications. However, by this definition, the conclusions in another systematic review [22] of another controversial oncologic advancement (proton beam therapy) would indicate that this technology is cost-effective for all evaluated tumors, which was not the conclusion posited therein. Lastly, if the threshold increased to $150,000/QALY, it may be concluded that nivolumab (without PD-L1 testing) would be cost-effective for HNCs [25, 26], NSCLC [28], RCC [35], and bladder cancer [36].

In addition to the major elements of careful patient selection and WTP thresholds, there are several other methods to improve CE profiles of ICIs. First, as elucidated by Matter-Walstra and colleagues, further investigation into reducing ICI dosing and duration is imperative [29]. As a result, evaluating whether clinical outcomes are compromised by altering these parameters would substantially influence CE and should ideally be a major focus of prospective investigation. Second, it is often overlooked that (although not a guarantee) time could cause prices of technologies or drugs to naturally decrease. In the drug development realm, increasing competition by means of generating biosimilars or generics has been extensively outlined [52–55]. Third, it is imperative to address quality of life (QOL) endpoints of a particular ICI, particularly because economic systems and payers are increasingly utilizing QOL to evaluate the “value” of funding new and expensive therapeutic approaches [56]. Fourth, although difficult in a free market economy, some have postulated that governmental negotiation of drug prices may save billions of healthcare dollars [52, 57, 58]; others have even advocated importing of drugs from abroad and increasing tax breaks for pharmaceutical companies that broaden “drug donation” programs [59].

Another point of emphasis in evaluating CE studies of ICIs is the large lack of long-term follow-up data from the original clinical trials on which the CE calculations are based. This is an extremely important notion, not only because the results of CE studies are heavily influenced by survival figures, but also because a purported benefit for immunotherapy lies in the durability of response and potential for longer-term survival. Because short follow-up cannot adequately assess for these endpoints, future publication of long-term follow-up studies should ideally focus on outcomes and toxicities that occur in the longer-term setting (as well as the duration/continuation of therapy and salvage treatments), as these influence long-term costs. A difficulty with modeling long-term costs from short-term follow-up data lies in the methodologies utilized for survival extrapolation. Several complex algorithms may be employed to extrapolate survival; however, because CE investigations are often very sensitive to survival estimates, various extrapolation methods can produce markedly different long-term survival values and thus CE profiles (for example, Wan et al. [33] and McCrea et al. [34]). Although multiple studies [25, 26, 36] utilized 3–5 year horizons given the difficulty in extrapolating OS from trials (3–4 years follow-up) to a CE investigation (time horizon of 20–30 years), the primary disadvantage is potentially missing long-term costs (the counter-argument, however, is primarily that most patients with metastatic cancers do not survive longer than 5 years, a paradigm further challenged by durable disease control and long-term survival recently reported with ICIs [60]).

There are limitations to every CE analysis, not only related to particular WTP values, but also because no study can entirely account for every possible cost-related factor or uncertainty in the studied factors. For example, it is impractical for studies to take into account global economic/market forces, practice/referral patterns, or reimbursement of a particular insurance company. CE and WTP also do not take into account other fiscally-related factors, such as the financial impact on patients and supportive care providers, receipt of other expensive oncologic therapies, and the ability to return to work and/or contribute to the workforce/economy. The proportion of total costs related to acute or delayed side effects is also a concern that has been largely not addressed in existing CE studies, largely owing to a lack of long-term follow-up. Applicability is also limited for several additional reasons, in addition to the country/economic system where the particular CE study was performed. First, very few studies varied and/or specifically addressed the dose, duration, and/or frequency of ICIs [29]. It is currently unknown when to stop ICI therapy provided a patient responds early and/or maintains stable disease; it is also clear that patients who prematurely stop ICIs may continue to respond. Additionally, because nivolumab is now approved for delivery every 4 weeks (instead of two) [61], the given CE studies may become less applicable going forward. Because ongoing clinical trials (NCT02713867, NCT02714218) are beginning to incorporate these regimens, cost analyses from those publications are also anticipated. Collectively, these valid concerns are notable barriers to understanding CE [62], and hence conclusions of any of the cited CE studies (and thus, this systematic review) could differ based on these variables alone. Second, most (but not all [43]) studies only factored in grade ≥ 3 toxicities, but not grade 1–2 events. Although cost estimates are not expected to be highly altered by including the latter, they often are associated with additional medications and possibly more frequent physician visits, all of which have associated costs. Third, critical evaluation of any CE investigation’s conclusions must be contextualized that numerous studies are co-authored by personnel of pharmaceutical companies [28, 31, 32, 34, 37, 39, 41, 42]. Fourth, there were only a limited number of studies from the initial literature search included in the final analysis (20 of 453), which may be associated with additional unforeseen biases. Lastly, CE studies cannot be extrapolated to other clinical settings and/or patients (e.g. nivolumab/ipilimumab for NSCLC, or nivolumab versus pembrolizumab for GUCs). Despite these known limitations, however, it is asserted that CE analyses are not intended to be exact (and should not be interpreted as such), but rather provide general economic estimates that must always be corroborated by replicative publications before firm conclusions are made. Additionally, because CE studies are heavily influenced by available clinical data, future publications of pivotal trials may also rapidly change the conclusions of this systematic review.

## Conclusions

To address the escalating costs of cancer care, it is crucial to provide evidence-based justification for promising but expensive therapeutic approaches such as ICIs. This systematic review, using an overall reference WTP threshold of $100,000/QALY, demonstrates several important findings. First, nivolumab is not cost-effective for recurrent/metastatic HNCs. Next, pembrolizumab is cost-effective for recurrent/metastatic NSCLC; although not the case for nivolumab, applying PD-L1 cutoffs may result in adequate CE. Regarding GUCs, most data for nivolumab or pembrolizumab would not result in CE using a WTP threshold of $100,000/QALY. Contrary to ipilimumab, either nivolumab or pembrolizumab are cost-effective for melanoma. Despite these conclusions, it cannot be overstated that careful patient selection is absolutely critical for CE; moreover, future publication of CE investigations and clinical trials (as well as longer follow-up of existing data) could substantially alter the conclusions of this analysis.

## References

[1] American Cancer Society. Immune Checkpoint Inhibitors to Treat Cancer. https://www.cancer.org/treatment/treatments-and-side-effects/treatment-types/immunotherapy/immune-checkpoint-inhibitors.html. Accessed 4 Apr 2018.

[2] Robert C, Thomas L, Bondarenko I (2011). Ipilimumab plus dacarbazine for previously untreated metastatic melanoma. N Engl J Med.

[3] Robert C, Long GV, Brady B (2015). Nivolumab in previously untreated melanoma without BRAF mutation. N Engl J Med.

[4] Robert C, Schachter J, Long GV (2015). Pembrolizumab versus Ipilimumab in Advanced Melanoma. N Engl J Med.

[5] Weber JS, D’Angelo SP, Minor D (2015). Nivolumab versus chemotherapy in patients with advanced melanoma who progressed after anti-CTLA-4 treatment (CheckMate 037): a randomised, controlled, open-label, phase 3 trial. Lancet Oncol.

[6] Brahmer J, Reckamp KL, Baas P (2015). Nivolumab versus docetaxel in advanced squamous-cell non-small- cell lung Cancer. N Engl J Med.

[7] Borghaei H, Paz-Ares L, Horn L (2015). Nivolumab versus docetaxel in advanced nonsquamous non-small- cell lung Cancer. N Engl J Med.

[8] Larkin J, Chiarion-SIleni V, Gonzalez R (2015). Combined Nivolumab and Ipilimumab or monotherapy in untreated melanoma. N Engl J Med.

[9] Motzer RJ, Escudier B, McDermott DF (2015). Nivolumab versus Everolimus in advanced renal-cell carcinoma. N Engl J Med.

[10] Herbst RS, Baas P, Kim DW (2016). Pembrolizumab versus docetaxel for previously treated, PD-L1- positive, advanced non-small- cell lung cancer (KEYNOTE-010): a randomised controlled trial. Lancet.

[11] Fehrenbacher L, Spira A, Ballinger M (2016). Atezolizumab versus docetaxel for patients with previously treated non-small-cell lung cancer (POPLAR): a multicentre, open-label, phase 2 randomised controlled trial. Lancet.

[12] Reck M, Rodriguez-Abreu D, Robinson AG (2016). Pembrolizumab versus chemotherapy for PD-L1–positive non–small-cell lung Cancer. N Engl J Med.

[13] Ferris RL, Blumenschein G, Fayette J (2016). Nivolumab for recurrent squamous-cell carcinoma of the head and neck. N Engl J Med.

[14] Bellmunt J, de Wit R, Vaughn DJ (2017). Pembrolizumab as second-line therapy for advanced urothelial carcinoma. N Engl J Med.

[15] Wolchok JD, Chiarion-Sileni V, Gonzalez R (2017). Overall survival with combined Nivolumab and Ipilimumab in advanced melanoma. N Engl J Med.

[16] Antonia SJ, Villegas A, Daniel D (2017). Durvalumab after Chemoradiotherapy in stage III non–small-cell lung Cancer. N Engl J Med.

[17] Patel MR, Ellerton J, Infante JR (2018). Avelumab in metastatic urothelial carcinoma after platinum failure (JAVELIN solid tumor): pooled results from two expansion cohorts of an open-label, phase 1 trial. Lancet Oncol.

[18] Mariotto AB, Yabroff KR, Shao Y, Feuer EJ, Brown ML (2011). Projections of the cost of cancer care in the United States: 2010–2020. J Natl Cancer Inst.

[19] Schnipper LE, Davidson NE, Wollins DS (2016). Updating the American Society of Clinical Oncology value framework: revisions and reflections in response to comments received. J Clin Oncol.

[20] Woods B, Sideris E, Palmer S, Latimer N, Soares M. NICE DSU Technical Support Document 19: Partitioned Survival Analysis for Decision Modelling in Health Care: A Critical Review. http://scharr.dept.shef.ac.uk/nicedsu/wp-content/uploads/sites/7/2017/06/Partitioned-Survival-Analysis-final-report.pdf. Accessed 4 Mar 2018.

[21] Moher D, Liberati A, Tetzlaff J, Altman DG, PRISMA Group (2010). Preferred reporting items for systematic reviews and meta-analyses: the PRISMA statement. Int J Surg.

[22] Verma V, Mishra MV, Mehta MP (2016). A systematic review of the cost and cost-effectiveness studies of proton radiotherapy. Cancer.

[23] Guglieri-Lopez B, Perez-Pitarch A, Porta Oltra B (2016). Effectiveness, toxicity, and economic evaluation of ipilimumab for the treatment of patients with metastatic melanoma in the Spanish outpatient setting. Anti-Cancer Drugs.

[24] Norum J, Antonsen MA, Tollali T (2017). Pembrolizumab as second-line therapy in non-small cell lung cancer in northern Norway: budget impact and expected gain-a model-based analysis. ESMO Open.

[25] Ward MC, Shah C, Adelstein DJ (2017). Cost-effectiveness of nivolumab for recurrent or metastatic head and neck cancer. Oral Oncol.

[26] Zargar M, McFarlane T, Chan KKW, Wong WWL (2018). Cost-effectiveness of Nivolumab in recurrent metastatic head and neck squamous cell carcinoma. Oncologist.

[27] Tringale Kathryn R, Carroll Kate T, Zakeri Kaveh, Sacco Assuntina G, Barnachea Linda, Murphy James D (2017). Cost-effectiveness Analysis of Nivolumab for Treatment of Platinum-Resistant Recurrent or Metastatic Squamous Cell Carcinoma of the Head and Neck. JNCI: Journal of the National Cancer Institute.

[28] Goeree R, Villeneuve J, Goeree J (2016). Economic evaluation of nivolumab for the treatment of second-line advanced squamous NSCLC in Canada: a comparison of modeling approaches to estimate and extrapolate survival outcomes. J Med Econ.

[29] Matter-Walstra K, Schwenkglenks M, Aebi S (2016). A cost-effectiveness analysis of Nivolumab versus docetaxel for advanced nonsquamous NSCLC including PD-L1 testing. J Thorac Oncol.

[30] Aguiar PN, Perry LA, Penny-Dimri J (2017). The effect of PD-L1 testing on the cost-effectiveness and economic impact of immune checkpoint inhibitors for the second-line treatment of NSCLC. Ann Oncol.

[31] Huang M, Lou Y, Pellissier J (2017). Cost-effectiveness of pembrolizumab versus docetaxel for the treatment of previously treated PD-L1 positive advanced NSCLC patients in the United States. J Med Econ.

[32] Huang M, Lou Y, Pellissier J (2017). Cost effectiveness of Pembrolizumab vs. standard-of-care chemotherapy as first-line treatment for metastatic NSCLC that expresses high levels of PD-L1 in the United States. Pharmacogenomics.

[33] Wan XM, Peng LB, Ma JA, Li YJ (2017). Economic evaluation of Nivolumab as a second-line treatment for advanced renal cell carcinoma from US and Chinese perspectives. Cancer.

[34] McCrea C, Johal S, Yang S, Doan J (2018). Cost-effectiveness of nivolumab in patients with advanced renal cell carcinoma treated in the United States. Exp Hematol Oncol.

[35] Sarfaty M, Leshno M, Gordon N (2018). Cost effectiveness of Nivolumab in advanced renal cell carcinoma. Eur Urol.

[36] Sarfaty Michal, Hall Peter S., Chan Kelvin K.W., Virik Kiran, Leshno Moshe, Gordon Noa, Moore Assaf, Neiman Victoria, Rosenbaum Eli, Goldstein Daniel A. (2018). Cost-effectiveness of Pembrolizumab in Second-line Advanced Bladder Cancer. European Urology.

[37] Barzey V, Atkins MB, Garrison LP (2013). Ipilimumab in 2nd line treatment of patients with advanced melanoma: a cost-effectiveness analysis. J Med Econ.

[38] Curl P, Vujic I, van’t Veer LJ (2014). Cost-effectiveness of treatment strategies for BRAF-mutated metastatic melanoma. PLoS One.

[39] Bohensky MA, Pasupathi K, Gorelik A (2016). A cost-effectiveness analysis of Nivolumab compared with Ipilimumab for the treatment of BRAF wild-type advanced melanoma in Australia. Value Health.

[40] Oh A, Tran DM, McDowell LC (2017). Cost-effectiveness of Nivolumab-Ipilimumab combination therapy compared with monotherapy for first-line treatment of metastatic melanoma in the United States. J Manag Care Spec Pharm.

[41] Wang J, Chmielowski B, Pellissier J (2017). Cost-effectiveness of Pembrolizumab versus Ipilimumab in Ipilimumab-Naïve patients with advanced melanoma in the United States. J Manag Care Spec Pharm.

[42] Miguel LS, Lopes FV, Pinheiro B (2017). Cost effectiveness of Pembrolizumab for advanced melanoma treatment in Portugal. Value Health.

[43] Kohn CG, Zeichner SB, Chen Q (2017). Cost-effectiveness of immune checkpoint inhibition in BRAF wild-type advanced melanoma. J Clin Oncol.

[44] Meng Yang, Hertel Nadine, Ellis John, Morais Edith, Johnson Helen, Philips Zoe, Roskell Neil, Walker Andrew, Lee Dawn (2018). The cost-effectiveness of nivolumab monotherapy for the treatment of advanced melanoma patients in England. The European Journal of Health Economics.

[45] Firwana Belal, Sonbol Mohamad Bassam, Atrash Shebli, Murad M. Hassan, Makhoul Issam, Hutchins Laura Fulper, Mahmoud Fade A. (2016). Efficacy of immunotherapy in advanced melanoma: A network meta-analysis. Journal of Clinical Oncology.

[46] Li X, Wang J, Yao Y (2017). Comparative efficacy and safety of immune checkpoint inhibitor-related therapies for advanced melanoma: a Bayesian network analysis. Oncotarget.

[47] Carbone DP, Reck M, Paz-Ares L (2017). First-line Nivolumab in stage IV or recurrent non-small-cell lung Cancer. N Engl J Med.

[48] Passiglia F, Galvano A, Rizzo S (2018). Looking for the best immune-checkpoint inhibitor in pre-treated NSCLC patients: an indirect comparison between nivolumab, pembrolizumab and atezolizumab. Int J Cancer.

[49] Tan PS, Aguiar P, Haaland B, Lopes G (2018). Comparative effectiveness of immune-checkpoint inhibitors for previously treated advanced non-small cell lung cancer – a systematic review and network meta-analysis of 3024 participants. Lung Cancer.

[50] Neumann PJ, Cohen JT, Weinstein MC (2014). Updating cost effectiveness—the curious resilience of the $50,000-per-QALY threshold. N Engl J Med.

[51] Ubel PA, Hirth RA, Chernew ME (2003). What is the price of life and why doesn’t it increase at the rate of inflation?. Arch Intern Med.

[52] The National Academies of Sciences, Engineering, Medicine. http://www8.nationalacademies.org/onpinews/newsitem.aspx?RecordID=24946&_ga=2.124021834.1182824426.1512055576-585989377.1512055576. Accessed 6 Apr 2018.

[53] Siddiqui M, Rajkumar SV (2012). The high cost of Cancer drugs and what we can do about it. Mayo Clin Proc.

[54] Weintraub A. Forbes magazine: pharma & healthcare. One Biotech CEO’s Plan to Slash the Cost of Cancer Immunotherapy https://www.forbes.com/sites/arleneweintraub/2015/09/17/one-biotech-ceos-plan-to-slash-the-cost-of-cancer-immunotherapy/#43779b465431. Accessed 31 Mar 2018.

[55] Maute RL, Gordon SR, Mayer AT (2015). Engineering high-affinity PD-1 variants for optimized immunotherapy and immuno-PET imaging. Proc Natl Acad Sci.

[56] Verma Vivek, Simone Charles B, Mishra Mark V (2017). Quality of Life and Patient-Reported Outcomes Following Proton Radiation Therapy: A Systematic Review. JNCI: Journal of the National Cancer Institute.

[57] Gellad WF, Schneeweiss S, Brawarsky P, Lipsitz S, Haas JS (2008). What if the federal government negotiated pharmaceutical prices for seniors? An estimate of national savings. J Gen Intern Med.

[58] Conti RM, Rosenthal MB (2016). Pharmaceutical policy reform — balancing affordability with incentives for innovation. N Engl J Med.

[59] Kantarjian H, Steensma D, Sanjuan JR, Elshaug A, Light D (2014). High Cancer drug prices in the United States: reasons and proposed solutions. J Oncol Pract.

[60] Gettinger Scott, Horn Leora, Jackman David, Spigel David, Antonia Scott, Hellmann Matthew, Powderly John, Heist Rebecca, Sequist Lecia V., Smith David C., Leming Philip, Geese William J., Yoon Dennis, Li Ang, Brahmer Julie (2018). Five-Year Follow-Up of Nivolumab in Previously Treated Advanced Non–Small-Cell Lung Cancer: Results From the CA209-003 Study. Journal of Clinical Oncology.

[61] Zhao Xiaochen, Ivaturi Vijay, Gopalakrishnan Mathangi, Shen Jun, Feng Yan, Statkevich Paul, Richards Eric, Rashford Michelle, Goodman Vicki, Gobburu Joga, Bello Akintunde, Roy Amit, Agrawal Shruti (2017). Abstract CT101: A model-based exposure-response (E-R) assessment of a nivolumab (NIVO) 4-weekly (Q4W) dosing schedule across multiple tumor types. Cancer Research.

[62] Verma Vivek (2018). Economic sustainability of immune-checkpoint inhibitors: the looming threat. Nature Reviews Clinical Oncology.

